# Transcriptional activation of the *CXCL* gene family locus in mature human sperm in the presence of female-derived reproductive fluid

**DOI:** 10.1098/rspb.2025.3286

**Published:** 2026-10-21

**Authors:** Tanja Turunen, Marjo Malinen, Sami Heikkinen, Merja Heinäniemi, Stephen A. Krawetz, Grace M. Swanson, Enni Linnavirta, Pia Allinen, Liisa Kuusipalo, Jukka Kekäläinen

**Affiliations:** ^1^ Department of Environmental and Biological Sciences, University of Eastern Finland, Joensuu, Finland; ^2^ Department of Physiology and Pharmacology, Karolinska Institutet, Stockholm, Sweden; ^3^ Department of Forestry, Food Technology, and Environmental Technology, South-Eastern Finland University of Applied Sciences, Kouvola, Finland; ^4^ Institute of Biomedicine, University of Eastern Finland, Kuopio, Finland; ^5^ Department of Obstetrics and Gynecology, Center for Molecular Medicine and Genetics, C.S. Mott Center for Human Growth and Development, Wayne State University School of Medicine, Detroit, MI, USA; ^6^ Center for Molecular Medicine and Genetics, Wayne State University School of Medicine, Detroit, MI, USA; ^7^ Ovumia Kuopio, Kuopio, Finland; ^8^ North Karelia Central Hospital, Joensuu, Finland

**Keywords:** sperm RNA biology, follicular fluid, sperm, fertilization, gene expression

## Abstract

Mature sperm are widely considered as transcriptionally inactive cells owing to the tight compaction of their chromatin with protamine. However, sperm cells have been demonstrated to retain RNA polymerase subunits, chromatin remodellers, transcription factors, and parts of their chromatin remain in a less condensed, nucleosome-bound configuration. Consequently, some studies have suggested that sperm may be transcriptionally more active than previously assumed. Here, we tested this possibility by exposing mature human sperm to follicular fluid (FF), a bioactive liquid surrounding the oocyte. Using RNA sequencing, we first observed that FF exposure alters the sperm transcriptome. To resolve whether the observed upregulation of selected candidate genes resulted from the transcriptional activation of these genes in sperm, we labelled newly synthesized RNAs with 5-ethynyl uridine. Our results suggest that FF may trigger *de novo* transcription of sperm *CXCL8* and *CXCL3* genes and indicate that some follicular-fluid-derived transcripts are selectively transferred into sperm. Collectively, these findings suggest that specific sperm genes may become transcriptionally active in the presence of biologically relevant stimuli, potentially preparing sperm for fertilization and early embryo development. Furthermore, we envisage that activated sperm genes can have novel implications for the deeper mechanistic understanding of sexual selection.

## 1. Introduction

Mature sperm are widely recognized as transcriptionally silent cells (e.g. [1]), which have been thought to result from the tight compaction of most of their DNA with protamines. However, in humans, approximately 15% of the sperm DNA remains in a more relaxed, nucleosome-bound configuration, where the DNA is wrapped around histones [2–4]. These protamine-bound and nucleosomal DNA regions are organized into two compartments that resemble the hetero- and euchromatin structure in the transcriptionally active somatic cells [5–8]. Within the nucleosomal chromatin, mature sperm cells exhibit both active (e.g. H3K4me3 and H3K4me1) and repressive (e.g. H3K27me3) histone post-translational modifications that are non-randomly distributed across the sperm genome [9–15]. Furthermore, the coexistence of active histone modifications (H3K4me1 or H3K4me3) with repressive H3K27me3 [16,17] probably potentiates the corresponding genomic regions, termed bivalent chromatin, for a rapid transcriptional activation. Mature sperm cells also retain subunits of RNA-synthesizing enzymes (RNA polymerases), transcription factors, and chromatin remodellers, and they are capable of RNA splicing [18], all of which are typically associated with transcriptional regulation and active transcription. Therefore, several authors have speculated that sperm may be transcriptionally more active than has traditionally been assumed [19–22]. Supporting this possibility, Naz [23] found indirect signs of transcriptional activity in sperm during capacitation. However, Grunewald *et al.* [1] observed no *de novo* RNA synthesis in mature sperm when testing for the incorporation of radio-labelled nucleotides in sperm RNA. Thus, unequivocal experimental evidence for sperm RNA synthesis is lacking.

Sperm are unable to fertilize oocytes immediately after ejaculation and thus need to undergo major physiological changes in the female reproductive tract (FRT) to acquire fertilization capacity [24,25]. In the FRT, sperm are guided towards the oocyte by factors called chemoattractants (chemotaxis) that are secreted by the oocyte and surrounding cumulus cells [26]. Follicular fluid (FF) is a bioactive fluid surrounding the oocyte in an ovarian follicle, and a rich source of sperm chemoattractants and other chemical factors, which are known to modify sperm motility and swimming behaviour (e.g. [27–29]). During ovulation, FF along with its chemical factors are emanated into the oviduct (e.g. [30]). FF also contains a wide array of proteins, amino acids, extracellular RNAs, and transcription factors [31] that can be transferred and incorporated into the sperm cells, possibly via exosomes or other extracellular vesicles [32]. Importantly, it has been envisaged that the normally dormant transcriptional machinery of the sperm could be reactivated in the presence of biologically relevant factors [19,20], which can be expected to be particularly abundant in FF.

Here, we test the possibility that FF activates gene expression in the mature human sperm. By using RNA-sequencing (RNA-seq) and subsequent identification of nascent transcripts by 5-ethynyl uridine (EU) labelling combined with polymerase chain reaction (PCR), our results indicate that FF enhances sperm RNA content both by inducing active messenger RNA (mRNA) transcription in sperm and by delivering specific transcripts into sperm. Together, our findings suggest that biologically relevant conditions that sperm experience before fertilization in the FTR must be considered to fully understand sperm's transcriptional capacity (cf. [33]).

## 2. Results

### (a) Follicular fluid and sperm RNAs

Semen samples were collected from three males, and mature sperm cells separated from each sample were equally divided into three treatment groups (nine sperm subsamples in total): untreated control, 0 h; sperm washing solution (SW)-treated, 3 h and FF-treated, 3 h (figure 1A). First, to investigate the effect of FF on sperm performance, we measured sperm curvilinear swimming velocity (VCL) in SW-treated and FF-treated sperm samples at two time points (30 and 180 min). We found that at both the time points, VCL was higher in the FF-treated samples (30 min: *F*_1,1964_ = 29.66, *p* < 0.001; 180 min: *F*_1,1386_ = 45.58, *p* < 0.001; figure 1A,B), demonstrating that FF treatment had an overall positive effect on sperm swimming velocity.

**Figure 1.**
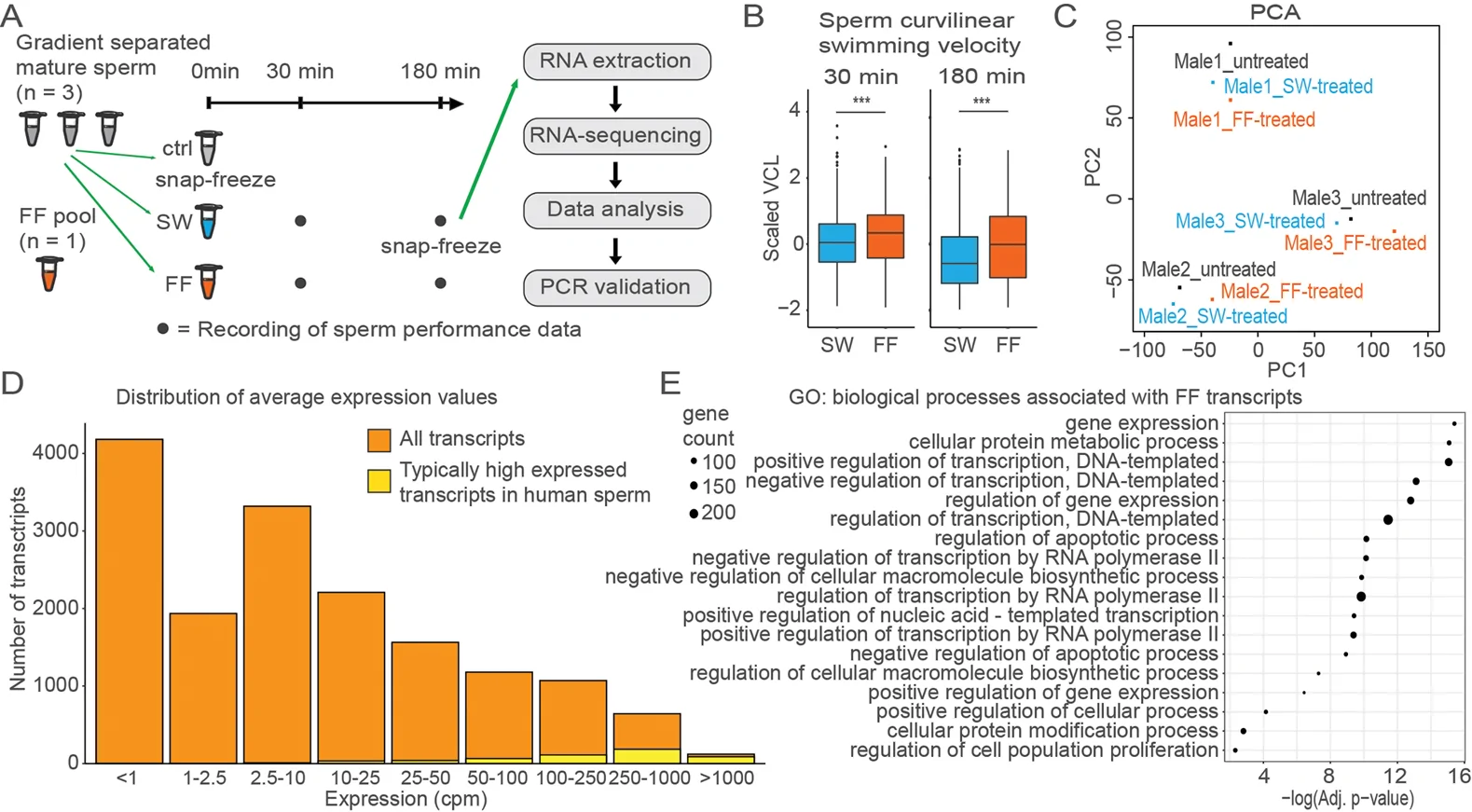
RNA-sequencing of human sperm and FF samples. (A) Schematic of the experimental set-up and workflow. (B) Sperm curvilinear swimming velocity (VCL) in SW- and FF-treated samples measured at 30 and 180 min time points. (C) Principal component analysis (PCA) of sperm RNA-seq samples. (D) Distribution of the average expression of mRNAs in the untreated sperm samples and comparison of highly expressed transcripts between the present study (orange bars) and Sendler *et al.* [34] (yellow bars). (E) Gene ontology analysis results showing the top biological processes enriched among mRNAs present in FF (raw read count > 20). Ctrl, untreated control sperm; SW, sperm washing solution-treated sperm; FF, follicular fluid-treated sperm. In the box plots, the lower and upper box boundaries represent the 25th and 75th percentiles, respectively, and the line inside the box represents the median. Lower and upper error lines indicate 1.5 × interquartile range, and dots indicate data points falling outside this range.

Then, we isolated sperm RNA and each of the above-mentioned nine sperm subsamples was subjected to transcriptome-wide RNA-seq. In line with earlier observations on the heterogeneity of human sperm transcriptome [35], donor identity was the predominant source of variance in principal component analysis (figure 1C; see also the supplementary material, figure S1A for pairwise correlation analysis of all individual sperm RNA samples). Nevertheless, SW and FF treatments showed subtle but distinct shifts in RNA composition within individuals. To validate the quality of our RNA-seq data, the expression of protein-coding sperm transcripts in each untreated control sperm sample was compared to the gene expression profiles provided by the genotype-tissue expression program (GTEx) [36]. The top 1000 genes expressed in the untreated control sperm samples showed expectedly a high positive linear correlation with the testis sample in GTEx data (Pearson correlation coefficients varied between 0.731 and 0.733; supplementary material, figure S1B). In comparison with all the other tissues, Pearson correlation coefficients varied from −0.014 to −0.045. Then, transcripts in our untreated control sperm data were grouped based on their mean expression and compared to previous sperm RNA-seq data [34]. Approximately 80% of the typically highly expressed human sperm mRNAs reported by Sendler *et al.* corresponded to the highly expressed transcripts observed in this study (counts per million > 50; figure 1D).

In addition to sperm RNA content, we characterized the RNA profile of the FF pool of five healthy women that was used in our FF treatments. Although the concentration of FF RNA was relatively low, we identified 1396 protein-coding genes, with expression levels of more than 20 raw read counts (supplementary material, table S1). Gene ontology analysis primarily highlighted biological processes associated with DNA-templated transcription and regulation of transcription by RNA polymerase II (figure 1E; supplementary material, table S2).

### (b) Follicular fluid treatment alters sperm RNA profile

Differential expression (DE) analysis between (i) untreated (0 h) and SW-treated (3 h); (ii) untreated (0 h) and FF-treated (3 h); and (iii) SW-treated (3 h) and FF-treated (3 h) sperm samples were carried out. Based on the statistically significant changes in their expression levels, DE genes (DEGs) were classified into upregulated and downregulated genes. With downregulated DEGs, we refer to all genes that showed decreased expression in the above-mentioned treatments. (i) The untreated versus SW-treated comparison revealed 137 DEGs, all of which were downregulated in the SW-treated sperm (adjusted *p*-value < 0.05 and logFC > |0.75|; supplementary material, table S3). (ii) The untreated versus FF-treated sperm comparison identified 163 downregulated DEGs and 30 upregulated DEGs in the FF-treated sperm (adjusted *p*-value < 0.05 and logFC > |0.75|; figure 2A; supplementary material, table S4). Finally, (iii) the SW- versus FF-treated sperm samples revealed one downregulated DEG and 17 upregulated DEGs in the FF-treated sperm (adjusted *p*-value < 0.05 and logFC > |0.75|; figure 2B; supplementary material, table S5).

**Figure 2.**
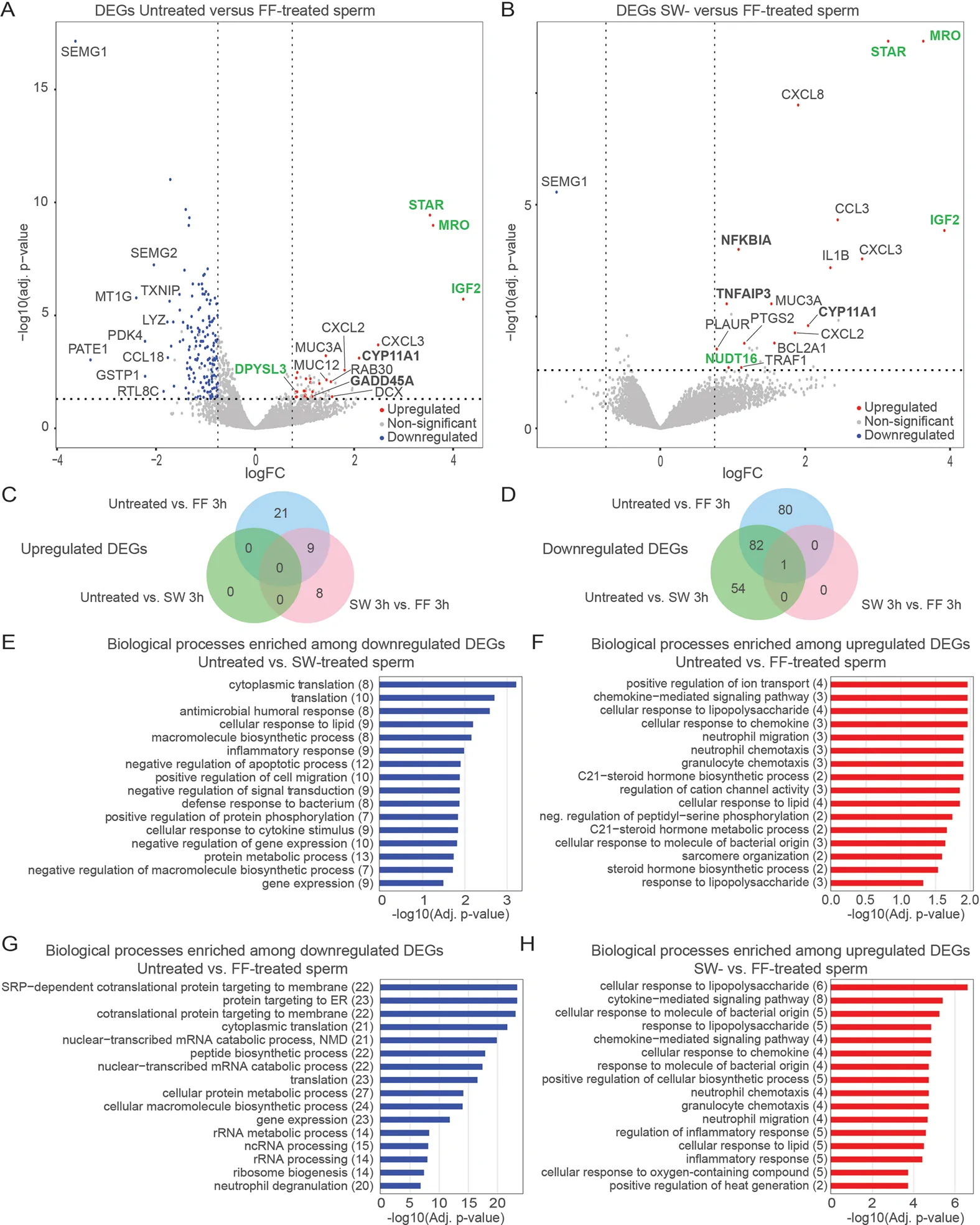
Differential expression analysis of RNA-sequencing data. (A) Volcano plot of differentially expressed genes (DEGs) identified between untreated versus FF-treated sperm samples (adjusted *p*-value < 0.05 and logFC > |0.75|). Gene names are shown for the 10 most significant DEGs and for those upregulated DEGs that were expressed in the FF sample (in bold). (B) Volcano plot of DEGs identified between SW-treated versus FF-treated samples (adjusted *p*-value < 0.05 and logFC > |0.75|). All gene names of significant findings are shown, as well as those upregulated DEGs that were expressed in the FF sample (in bold). In panels (A) and (B), blue spots indicate significant downregulation, and red spots indicate significant upregulation. Gene names further highlighted in green font were among the top 5% abundant transcripts in the FF sample. (C) Overlaps between all upregulated DEGs and (D) between all downregulated DEGs. Gene ontology analysis results showing the top biological processes (E) among the downregulated DEGs identified in untreated versus SW-treated samples. Gene ontology analysis results showing the top biological processes among (F) upregulated DEGs and (G) among the downregulated DEGs identified in panel (A), as well as (H) among the upregulated DEGs identified in panel (B). Blue bars (E,G) indicate results of downregulated DEGs, and red bars (F,H) indicate results of upregulated DEGs. Top gene ontology biological process terms with adjusted *p*-value < 0.05 (Benjamini–Hochberg correction method for multiple hypothesis testing) and a minimum of 5% of input genes are shown. The number of genes in each gene ontology term is shown in brackets after the term (E–H).

Nine DEGs were upregulated in both the untreated versus FF-treated and SW-treated versus FF-treated comparisons (figure 2C). Of the downregulated DEGs, 83 were consistently downregulated in untreated versus FF-treated and untreated versus SW-treated comparisons (figure 2D), i.e. the expression of these genes decreased regardless of the treatment. By contrast, 54 and 80 genes were exclusively downregulated in the untreated versus SW-treated sperm and untreated versus FF-treated comparisons, respectively. Additionally, the expression of six genes (*TNFAIP3*, *NFKBIA*, *CXCL8*, *CCL3*, *PTGS2* and *IL1B*) decreased in untreated versus SW-treated, but was upregulated in the SW-treated versus FF-treated comparison, suggesting that the observed upregulation of these genes could potentially in part result from mRNA degradation over time in SW-treated sperm rather than FF-induced upregulation. Gene lists of the comparisons presented in the Venn diagrams are reported in the supplementary material, table S6. Gene ontology analysis of the DEGs in the untreated versus SW-treated comparison revealed biological processes related to translation, signal transduction and immune functions (figure 2E; supplementary material, table S7). Untreated versus FF-treated comparison revealed that, e.g. chemokine signalling-, ion transport- and steroidogenesis-related biological processes were enriched among the upregulated DEGs, whereas those related to RNA processing, translation and protein targeting (to the endoplasmic reticulum or membrane) were enriched among the downregulated DEGs (figure 2F,G; supplementary material, tables S8 and S9). In the SW- versus FF-treated sperm comparison, in addition to chemokine signalling, cytokine signalling and several other immunological processes were enriched among the upregulated genes (figure 2H; supplementary material, table S10).

### (c) The *CXCL* gene family locus is transcriptionally activated in human mature sperm

To evaluate whether the FF-induced upregulation of DEGs could reflect *de novo* RNA transcription by sperm or selective transfer of mRNAs from FF into sperm, we compared the presence of upregulated DEGs (supplementary material, tables S3–S5) in the transcriptome of the pooled FF sample (supplementary material, table S1). We observed that six of the 30 upregulated DEGs (in the untreated versus FF-treated comparison) and seven of the 17 DEGs (in the SW- versus FF-treated comparison) were present in FF (shown in bold in figure 2A,B). Three common DEGs, *DPYSL3* in the earlier mentioned comparison and *NUDT16* in the latter comparison, were found among the top 5% abundant transcripts in FF (highlighted in green in figure 2A,B). This suggests that the increase in their expression could be owing to *de novo* transcription or potential selective transfer from FF into sperm (see below).

Among the upregulated DEGs, we identified several *CXCL* family genes located in the CXC chemokine locus on chromosome 4 ([37]; figure 3A). Upregulation of *CXCL2* and *CXCL3* was significant and *CXCL8* marginally significant (adjusted *p*-value = 0.052) in the untreated versus FF-treated comparison. Moreover, all these genes were upregulated in the SW- versus FF-treated comparison (adjusted *p*-value < 0.05). We selected *CXCL3* and *CXCL8*, which were not expressed in the pooled FF sample, and *IGF2* (chromosome 11), which is among the most expressed genes in the pooled FF sample, for nascent RNA capture analysis. Snapshots of the signal tracks within the genomic loci of these three selected genes show consistent patterns in all individuals, in agreement with the DE gene analysis results (three sperm samples with three treatments) in figure 3B.

**Figure 3.**
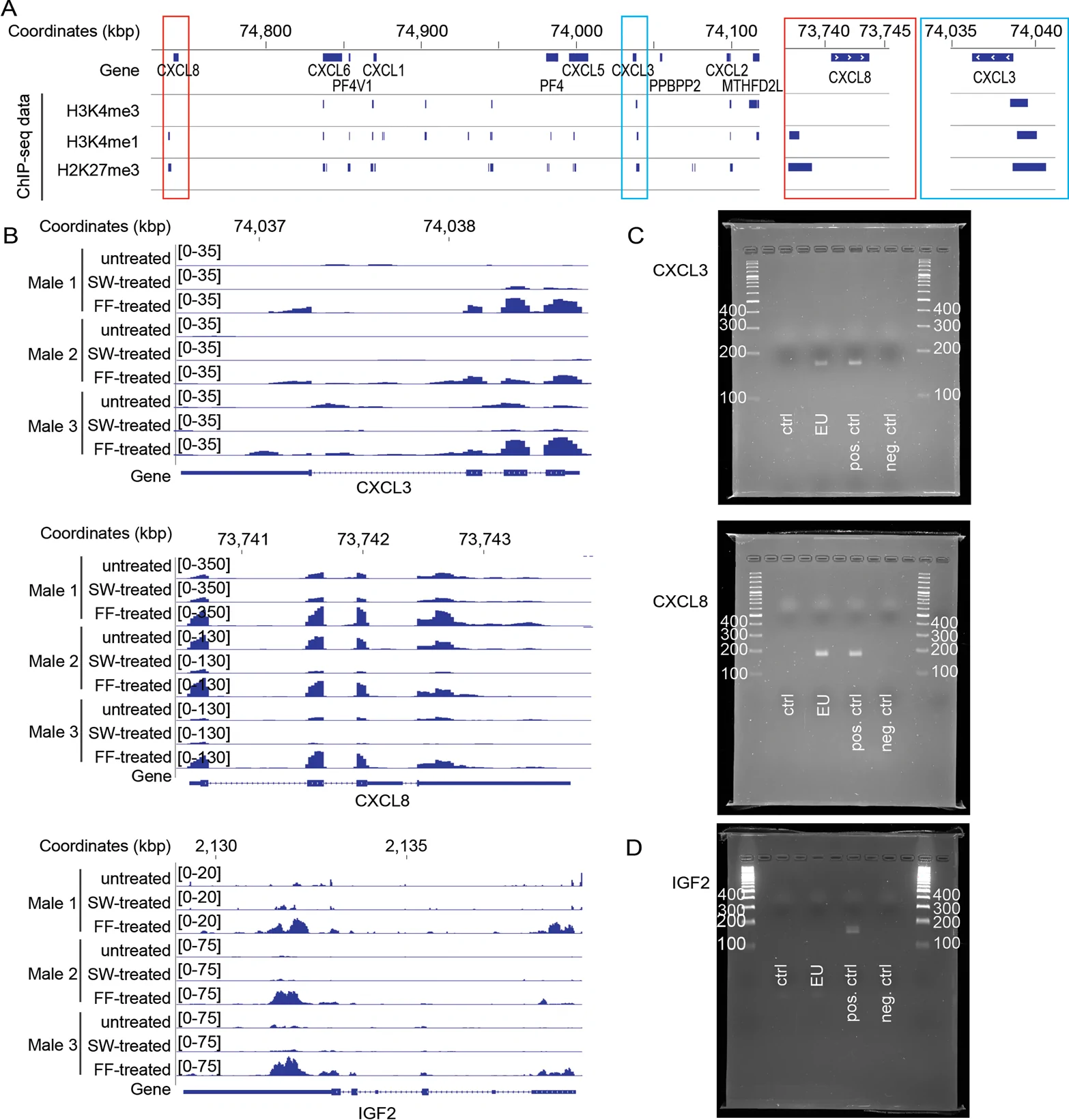
Identification of newly synthesized mRNAs upon FF exposure. (A) Signal tracks of epigenetic data [11,12] at the *CXCL* family locus (chr4: 73,583,165–74,165,210). The regions highlighted on the right correspond to the *CXCL8* (chr4: 73,736,631–73,745,350) and *CXCL3* (chr4: 74,035,434–74,041,053) gene regions. (B) Signal tracks of RNA-seq data at *CXCL3* (chr4: 2,128,977–2,139,587), *CXCL8* (chr4: 74,036,566–74,038,731) and *IGF2* (chr11: 73,740,460–73,743,883). PCR analyses for *CXCL3* and *CXCL8* (C) and *IGF2* (D) were performed using gene-specific exon-spanning primers, and PCR products were separated using agarose gel electrophoresis. Ctrl, sperm samples treated with FF pool in the absence of the labelled nucleotide, 5-ethynyl uridine, to control for any unspecific capture of RNAs; EU, sperm samples treated with FF pool in the presence of 5-ethynyl uridine to capture newly synthesized RNAs; pos. ctrl, cDNA synthesized from total RNA of an independent FF-treated sperm sample to show the expected size of PCR products and to account for potential non-specific PCR amplification that would show additional PCR products of unexpected size; neg. ctrl, a water sample to exclude any non-specific amplification.

To experimentally test whether the observed upregulation of *CXCL3* and *CXCL8* is caused by *de novo* RNA transcription in human sperm, we treated mature sperm of multiple males (two replicates: *n* = 5 and *n* = 4) with the FF pool for 3 h in the presence and absence of EU. EU is a labelled nucleotide that directly incorporates into newly synthesized RNAs upon treatment. Then, the sperm were lysed, the labelled nucleotide-containing RNAs were extracted, complementary DNA (cDNA) synthetized and subjected to end-point PCR analyses using gene-specific primers. When sperm was treated with FF in the presence of the labelled nucleotide, our results showed an amplified PCR product of *CXCL8* in both the replicates; however, for *CXCL3,* the signal strength of the amplified PCR product was less consistent between replicates, potentially owing to low expression level of the gene compared to *CXCL8* (see lane marked ‘EU’ in figure 3C for replicate 1 and the supplementary material, figure S2 for replicate 2. For differences in *CXCL3* and *CXCL8* expression levels, see figure 3B). Since the RNA content in sperm is relatively low compared to other cell types [38], *de novo* changes in sperm RNA levels can be expected to be of low magnitude, hindering their detection using PCR. The sperm samples treated with the FF pool in the absence of the labelled nucleotide were used to control for any unspecific capture of RNAs (lane marked ‘ctrl’ in figure 3C; supplementary material, figure S2). We also used cDNA synthesized from total RNA of an independent FF-treated sperm sample to show the expected size of PCR products and to account for potential non-specific PCR amplification that would show additional PCR products of unexpected size (lane marked ‘pos. ctrl’ in figure 3C; supplementary material, figure S2). Additionally, we included a water sample to ensure that no non-specific amplification was occurring (‘neg. ctrl’ in figure 3C; supplementary material, figure S2). To further exclude the possibility that results could represent nascent RNA production spontaneously occurring in the pooled FF sample, we incubated the FF pool with EU for 3 h and observed no amplified *CXCL3* and *CXCL8* PCR products. Together, these results provide robust evidence for active transcription of *CXCL8*, and preliminary evidence for *CXCL3*, in human mature sperm in response to FF treatment. This conclusion is also supported by the demonstrated absence of other cell type markers in extracted sperm RNA samples and assessment of potential FF factors associated with transcriptional activation (for detailed information, see the supplementary material, Methods and Results).

Next, we investigated the presence of nascent transcripts for *IGF2* in the same EU-treated sperm samples and observed no PCR product (figure 3D; supplementary material, figure S2). Additionally, we observed a downregulation of many highly abundant FF transcripts, such as *RPL3*, *RPL4* and *RPL13A*, in FF-treated sperm and found that FF transcript abundance did not generally predict the upregulation of these same transcripts in sperm (Spearman correlation coefficients: −0.189 and −0.055 for untreated versus FF 3 h and SW 3 h versus FF 3 h, respectively; supplementary material, figure S3A,B). Together, these findings indicate that the observed upregulation of *IGF2* originates from a nongenomic response, that is, a selective transfer of FF-borne mRNA into the sperm or sperm surface rather than from a contamination of FF-treated sperm samples by random FF mRNA (sperm-unincorporated FF mRNA left in washed sperm pellet).

Finally, to inspect whether the genes of interest show an overlap of active and repressive histone modifications, a bivalent chromatin state, in their close proximity, we complemented the RNA-seq and nascent RNA capture analyses by previously published chromatin immuno-precipitation followed by sequencing (ChIP-seq) data for H3K4me3, H3K4me1 and H3K27me3 collected from mature human sperm [11,12]. Interestingly, both *CXCL3* and *CXCL8* showed an overlap of active (*CXCL3*: both H3K4me3 and H3K4me1; *CXCL8:* H3K4me1) and repressive (H3K27me3) histone modifications closely upstream from their promoter regions (figure 3A), indicating that these genes show a bivalent state and are primed for rapid transcriptional activation in the presence of adequate biological stimuli. Instead, *IGF2* did not exhibit any of these histone modifications in their close vicinity. Since *IGF2* is present in FF, this finding supports our conclusion that it is most probably selectively transferred from FF into sperm.

## 3. Discussion

Sperm undergo final maturation after ejaculation into the FRT, where they become functionally capable of fertilizing an oocyte. The present results indicate that these maturational changes may involve selective transcriptional activation of specific sperm genes, in conjunction with transfer of FF-borne transcripts into the sperm. The upregulated genes in response to FF in sperm predominantly encoded factors related to chemokine and cytokine signalling, ion transport and steroidogenesis, while downregulated genes were mainly associated with translation and RNA processing. Regarding *de novo* transcription, our data showed strong evidence for *CXCL8* and preliminary evidence for *CXCL3* transcription in response to FF treatment, whereas *IGF2* seemed to be selectively transferred from FF into sperm. Collectively, these results may begin to resolve why somewhat less condensed, nucleosome-bound chromatin and several components of transcription machinery are present in sperm. Our results may also have novel implications for a deeper mechanistic understanding of fertilization and potentially many other processes, like priming the early embryo [15,39].

When released into the FRT, sperm experience a radical change in their external milieu and interact with somatic cells and secretions from the FRT. *In vitro* studies have shown that FF, a secretion of the FRT, has a major influence on sperm physiology and function [24,27,40]. FF and other FRT secretions contain numerous extracellular factors, many of which are transferred into sperm cells either directly or via extracellular vesicles [24,31,41]. Besides mediating interactions between the FRT and sperm, FF has several important regulatory functions in both the ovary and the oviduct, such as triggering changes in gene expression in cumulus cells, oviductal epithelial cells, and *in vitro* maturing oocytes [42–44]. Although these studies did not test the possibility of selective transfer of FF transcripts into these cells, their results suggest that some currently unknown FF factors may be capable of activating gene expression also in mature human sperm.

Along with the effects of FF, our results revealed that the used control solution (SW) caused downregulation in a total of 137 genes in comparison to untreated sperm. Since RNA composition of the untreated sperm represented the situation before the beginning of the sperm treatments (0 h) and SW-treated and FF-treated sperm were both treated for 3 h, it seems likely that RNA is either passively or actively degraded in SW-treated sperm during this time. This is important as SW-induced downregulation could potentially lead to misclassification of such downregulated genes as upregulated in the SW-treated versus FF-treated comparison. However, only six of these genes were found to be upregulated in SW-treated versus FF-treated sperm, indicating that at least 11 out of 17 upregulated genes in SW versus FF comparison were genuinely upregulated owing to FF treatment. Importantly, one of the six genes was *CXCL8*, which was later validated to be *de novo* transcribed in the presence of FF, which further strengthened this view.

Despite the fact that the majority of the sperm genome is tightly packaged by protamines, approximately 15% of the genome remains bound to nucleosomes [2–4]. Contrary to somatic cells, these more accessible chromatin regions in sperm tend to be located in the nuclear periphery [45,46]. This spatial localization, together with the more relaxed chromatin configuration, can be expected to make these chromatin regions as the first targets of oocyte-derived transcription activators (e.g. [5]). Accordingly, we show strong evidence for *de novo* transcription of *CXCL8* and less robust evidence for *CXCL3*, both of which exhibit a bivalent chromatin state of overlapping active and repressive histone modifications, in close proximity upstream from their promoter regions. These genes are located in chromosome 4, which is one of the most peripherally located chromosomes in human sperm [47]. By contrast, we did not observe *de novo* transcription of *IGF2* that is located more centrally within sperm nuclei [47] with no histone modifications close to its loci. Therefore, it seems plausible that some currently unknown FF factor(s) have access to the regulatory regions of *CXCL3* and *CXCL8*, consistent with their activation. Supporting this possibility, it has been demonstrated that many of the key components of the transcriptional machinery, such as several subunits of RNA polymerases and Mediator, histone deacetylases, histone demethylases and many transcription factors, are present in mature human sperm (e.g. [48]).

Even if the function of FF-induced transcription of *CXCL3* and *CXCL8* is currently unclear, we envisage that their upregulation in sperm may participate in regulating early embryo development. Accordingly, *CXCL3* is known to regulate the migration, invasion and proliferation of trophoblast cells [49], whereas the presence of *CXCL8* in the embryo culture medium positively associates with embryo developmental potential in *in vitro* fertilization patients [50]. In comparison, other studies have shown that changes in sperm transcriptome are associated with sperm motility and capacitation [21,51], indicating that the expression of *CXCL3* and *CXCL8* may also play a role in regulating sperm fertilization ability. However, further investigations are required to uncover the underlying mechanisms of FF-mediated transcriptional activation of sperm. Additionally, even if we conducted all appropriate negative controls and they looked as expected, we acknowledge that EU labelling of nascent RNA may sometimes lead to some non-specific pull-down of non-EU-labelled transcripts. Thus, we encourage future studies to replicate our experiment by using some alternative nascent RNA sequencing or labelling technologies. Furthermore, since the current understanding is that sperm RNAs are reflective of male and their offspring health [52–56], additional research is needed to clarify potential intergenerational effects of FRT-induced changes in sperm transcriptome.

After fertilization, sperm undergo nuclear decondensation in the ooplasm, and their protamines are rapidly removed from the chromatin. However, it has been shown both in humans [57] and in rams [58] that FF can cause sperm chromatin decondensation before fertilization. In the first stage, nuclear decondensation includes reduction of the disulphide bonds in protamines, which is known to be mediated by glutathione, heparan sulphate, protein disulphide isomerase-associated 3 (PDIA3) and nucleoplasmin family proteins 1–3 (NPM1, NPM2 and NPM3) [59–62]. Importantly, the first three substances as well as NPM1 have also been found in human FF [48,63,64]. Additionally, the use of human sperm that undergo faster decondensation has been associated with higher quality embryos following *in vitro* fertilization compared to sperm with a slower decondensation response [59]. Therefore, it is possible that FF-induced selective removal of protamines could play an important role in the activation of sperm gene expression and this way, start preparing sperm for fertilization and post-fertilization events [65].

Supporting this view, it has been shown that capacitated sperm exhibit increased disulphide bond reduction of protamines as well as increased levels of chromatin decondensation [58]. Furthermore, Péres-Cerezales *et al.* [66] demonstrated that the sperm subpopulation (‘selected sperm’) that enters the fallopian tube (i.e. potentially fertilizing sperm) has lower chromatin compaction and higher DNA integrity, and yields higher-quality embryos than unselected sperm. Together with the present findings, these results are consistent with the view that the FRT may have evolved mechanisms that allow females to select sperm for fertilization based on their RNA cargo and nascent RNA production. In line with this conclusion, a number of studies have demonstrated that mate choice also occurs at the level of the gametes, and that ‘gamete-mediated mate choice’ (GMMC) is often mediated by various female-derived reproductive secretions (reviewed by [67]). Furthermore, Kekäläinen [68] recently proposed that FF and other FRT secretions can mediate GMMC also at the level of individual sperm cells. All the sperm genes that are differentially expressed among individual mature sperm cells (either at the RNA or protein level) are potential targets of this emerging (haploid) selection process. Importantly, Bhutani *et al.* [69] have shown that at least one third of the transcripts are unequally shared through spermatid cytoplasmic bridges during spermatogenesis, supporting the possibility that GMMC at the level of individual sperm cells could occur. However, since we did not investigate gene expression heterogeneity of nascent RNAs (or proteins), future studies are needed to clarify the potential role of sperm gene expression in the process.

An important prerequisite for haploid selection is that sperm phenotype is associated with its RNA (or protein) composition and that sperm phenotype affects offspring phenotype and/or fitness. Importantly, along with Bhutani *et al.* [69], recent studies have found tentative evidence for both criteria (e.g. [70,71]). Together, these data raise the possibility that the FRT can select sperm for fertilization even at the level of individual spermatozoa. Therefore, selection during the haploid gametic stage may be more common than has previously been assumed [70] and could be driven by context-dependent gene expression [19]. However, further experimental research is required to test the validity of these assumptions and to understand the exact role of FF and other female-derived reproductive secretions in sperm epigenetic modifications.

In conclusion, our results suggest that FF may activate the expression of *CXCL* family genes in mature human sperm, and this way potentially shape the RNA payload for delivery to the egg. We propose that the transcriptional activation of the specific sperm genes may be an important part of the sperm maturation process within the FRT, potentially promoting sperm's preparation for fertilizing the oocyte and impacting early embryo development. Our results may begin to open the door for a deeper understanding for the presence of the key components of the transcriptional machinery, potentiated chromatin and transcriptional regulators in mature human sperm. Finally, our findings raise the possibility that sperm gene expression may be a more widespread phenomenon than previously considered, becoming only evident when investigated in the presence of biologically relevant stimuli.

## 4. Material and methods

### (a) Ethical approval and study participant details

The study was conducted in accordance with the Declaration of Helsinki and approved by the Ethics Committee, Hospital District of Northern Savo, Finland (decision number 77/2017). All methods were performed in accordance with the relevant guidelines and regulations. All participants in this study volunteered and signed the informed consent before enrolment. Male participants were recruited via public advertisements, and female participants were healthy egg donors who enrolled in the study via the private fertility clinic.

The semen samples for RNA-seq data collection fulfilled the World Health Organization criteria for reproductive age (29.05 ± 1.20 yr, mean ± standard error of the mean (s.e.m.)), normal semen volume, liquefaction time, pH, viscosity and sperm count (supplementary material, table S11) [72]. Semen samples for RNA-seq were donated by normal-weight, non-smoking men (*n* = 3) who reported no diseases, medications or history of infertility. Semen samples for subsequent nascent RNA validations included sperm samples of nine males without a history of infertility (age: 30.56 ± 1.50 yr, mean ± s.e.m.). All males were Caucasian. FF samples were obtained from five healthy oocyte donor women (age 26.0 ± 1.95 yr, mean ± s.e.m.).

### (b) Sample collection

All semen samples were provided by masturbation after 2–4 days of sexual abstinence. After the collection, samples were allowed to liquefy for 30 min at +37°C and were then processed immediately. To separate mature sperm from semen, all the above-mentioned semen samples were first layered over 40% and 80% discontinuous PureSperm gradients (Nidacon International AB, Mölndal, Sweden) and centrifuged at 300 *g* for 20 min at room temperature (RT). Cell pellets were then resuspended in 5 ml of PureSperm® Wash solution (Nidacon) and centrifuged at 500*g* for 10 min at RT.

After collection, FF samples were centrifuged at 500*g* for 10 min, and the supernatant was aliquoted and stored in liquid nitrogen (see [73] for detailed protocol). The five women's FF samples were later thawed, pooled and aliquoted, and restored in liquid nitrogen until use (see below).

### (c) Sperm treatments and recording of sperm motility

The gradient-washed, mature sperm samples from each of the three males were divided into three aliquots, which were kept and treated separately (nine aliquots in total, see below). One aliquot within each male was immediately pelleted, snap-frozen and stored at −80°C as an untreated control sample. The remaining aliquots were treated (1 : 1, vol : vol) for 3 h with either PureSperm® Wash solution (Nidacon) (SW-treated) or the above-mentioned FF pool (FF-treated) in 30–40 × 10^6^ cells ml^−1^ concentration at +37°C. These controls (untreated and SW-treated) were included to account for the changes in relation to baseline gene expression and to allow for distinguishing the effect of sperm incubation time from the effect of FF treatment on sperm gene expression. Sperm motility recordings and the corresponding statistical analyses are described in the supplementary material, Methods.

### (d) RNA extraction, RNA quality measures and sequencing data pre-processing

Large RNAs (> 200 bp) were extracted and purified from each individually treated sperm subsample and the pooled FF sample using the RNEasy mini kit (QIAGEN GmbH, Hilden, Germany) according to the manufacturer's recommendations and as previously described in [74–76]. To confirm the absence of genomic DNA contamination (sperm DNA or extracellular DNA), extracted RNA was off-column DNAse treated with RNase-Free DNase Set (QIAGEN GmbH, Hilden, Germany) using the manufacturer's instructions. An aliquot of each RNA sample was used to check the absence of DNA contamination using exon-spanning primers for the *PRM1* gene, as described in [74–76]. Thermal conditions in end-point PCR were as follows: 95°C for 10 min, (95°C for 20 s, 62°C for 20 s, 72°C for 20 s) 40 cycles and 72°C for 5 min. Images of agarose gels are shown in the supplementary material, figure S4A. DNAse treatment was repeated until no DNA contamination was observed in any of the RNA samples. To assess sperm sample purity, i.e. to exclude the possibility of contamination by certain somatic and testicular germ cells, we tested the absence of specific marker RNAs in sperm RNA samples (protein tyrosine phosphatase receptor type C (*PTPRC)* for leucocytes*, c-kit* for testicular germ cells, and E-cadherin (*CDH1)* for epithelial cells), as previously described in [77]. Thermal conditions in end-point PCR were as follows: 95°C for 10 min, (95°C for 20 s, primer-specific temperature for 20 s, 72°C for 20 s) 35 cycles and 72°C for 5 min. Images of agarose gels are shown in the supplementary material, figure S4B. All primers used are described in the supplementary material, table S12. Finally, the RNA integrity and size profiles were determined using Agilent 2100 Bioanalyzer (Agilent Technologies, Waldbronn, Germany). The mean RNA integrity number of our samples was 3.7 (±0.3 s.e.m.) and the majority of the RNA sizes ranged between 0.2 and 1.5 kb, which are typical for sperm RNA [78]. Sequencing library preparation, sequencing and both RNA-seq and ChIP-seq data pre-processing are described in detail in the supplementary material, Methods.

### (e) Differential expression analysis and comparison to previously collected data

Before the differential expression analysis, we selected nuclear protein-coding transcripts and compared the expression levels of untreated control sperm samples (*n* = 3) to previous sperm transcriptome data. In the comparison, we used the list of typical sperm transcripts [34] and divided transcripts into separate groups based on their expression level. Moreover, we calculated Pearson correlation of gene expression profiles of the top 1000 genes (based on our untreated control sperm RNA-seq samples) between our sperm RNA-seq samples and gene expression profiles in multiple tissues provided by the genotype-tissue expression program (GTEx). Differentially expressed protein-coding genes were calculated following the edgeR-limma-based DE analysis pipeline described in [79]. For detailed description of DE analysis workflow, see the supplementary material. Genes with an adjusted *p*-value of less than 0.05 (Benjamini–Hochberg method) and logFC > |0.75| were included in the downstream analysis. To ensure that DE analysis results are true positives, i.e. supported by changes in signals located to exons of identified DE genes, we manually examined all DE transcripts based on the signal tracks using the Integrative Genome Viewer. Transcripts in which the signals were based on expression of only one exon in genes with multiple exons or transcripts that showed a low signal-to-noise ratio throughout the gene body were excluded. Other bioinformatic quality measures, such as evaluation of the potential effect of other cell types on the sperm transcriptome, are explained in the supplementary material, Methods.

### (f) Gene ontology analysis

Up- and downregulated DEGs from each comparison and FF transcripts (raw read count > 20) were separately used as inputs to the enrichR tool [80]. Adjusted *p*-values were computed for all gene ontology terms using the Benjamini–Hochberg correction method for multiple hypothesis testing. Gene ontology terms with adjusted *p*-values < 0.05 were considered significant. Moreover, we required more than 5% of the input genes to be associated with the gene ontology term in analyses. Additionally, gene ontology terms associated with FF proteins [48] were analysed using the enrichR tool [80].

### (g) Data visualization

Results were visualized using the ggplot2 (v. 3.4.0), gplots (v.3.1.3), ComplexHeatmap (v. 2.14.0) and ggrepel (v. 0.9.2) R packages [81–83].

### (h) Nascent RNA capture followed by polymerase chain reaction

Mature sperm cells were separated from semen as described above. Then, we captured newly synthesized RNAs using the Click-iT® RNA capture assay (Invitrogen™, Thermo Fisher Scientific, Eugene OR USA) according to the manufacturer's instructions for the smallest RNA amount. The sperm of each of the nine males was divided into two aliquots, both of which were combined 1 : 1 (vol : vol) with the FF pool of five females. One aliquot was supplemented with 0.2 mM of EU, which incorporates into the newly synthesized RNA, whereas the other aliquot acted as a control sample (no EU added). The sample without EU treatment was used to exclude the possibility of a non-specific RNA capture-derived false-positive result. Both aliquots were incubated for 3 h and were then snap-frozen and stored at −80°C until RNA extraction. We performed the assay in two independent replicates. Replicate 1 included the mature sperm of five males (three of whom were the same individuals that participated in RNA-seq; see above) and replicate 2 included mature sperm from four additional male donors. To maximize the yield of nascent RNA, sperm samples (within each replicate) were pooled together before RNA extraction, which was performed as described above, and followed by nascent RNA capture according to the manufacturer's instructions. Nascent RNA from FF-treated sperm in the presence and absence of EU (referred to as EU and ctrl, respectively) as well as total RNA from an independent FF-treated sperm sample (pos. ctrl) were inputted to cDNA synthesis using the SuperScript® VILO^TM^ cDNA synthesis kit (Thermo Fisher Scientific, Carlsbad, CA, USA). Additionally, nuclease-free water was included in the PCR to control for any non-specific amplification or contamination (neg. ctrl). PCR was performed using gene-specific, exon-spanning primers (only aligning to the gene of interest based on a nucleotide BLAST analysis, primers listed in the supplementary material, table S12). Thermal conditions in end-point PCR were as follows: 95°C for 10 min, (95°C for 20 s, primer-specific temperature for 20 s, 72°C for 20 s) 40 cycles and 72°C for 5 min.

## Supplementary Material



## Data Availability

The RNA-seq dataset is provided through Gene Expression Omnibus under accession number GSE290246 [84]. Sperm performance data and pre-processed RNA-seq data as well as scripts for data pre-processing and downstream analyses are provided online via [85]. Other supplementary material is available online [86].
