# Supplementary material for: Transcriptional activation of the CXCL gene family locus in mature human sperm in the presence of female-derived reproductive fluid
Source: Proc Biol Sci. 2026 Oct 21;293(2081):20253286. doi: 10.1098/rspb.2025.3286 (PMC13682880; doi:10.1098/rspb.2025.3286)
Supplement: Supplementary file 1 [file rspb.2025.3286.zip › rspb.2025.3286/rspb20253286_si_001.pdf]

# Supplementary Material – Results, Methods, Figures

## Transcriptional activation of the CXCL gene family locus in mature human sperm in the presence of female-derived reproductive fluid


## Proceedings of the Royal Society B: Biological Sciences

## 10.1098/rspb.2025.3286



**Turunen, Tanja[1,2]; Malinen, Marjo[1,3]; Heikkinen, Sami[4]; Heinäniemi, Merja[4]; Krawetz, Stephen A.[5,6]; Swanson, Grace M.[6]; Linnavirta, Enni[1]; Allinen, Pia[7]; Kuusipalo, Liisa[8]; Kekäläinen, Jukka[1*]**

Author affiliations:
[1] University of Eastern Finland, Department of Environmental and Biological Sciences, Joensuu, North Karelia, Finland

[2] Karolinska Institutet, Department of Physiology and Pharmacology, Stockholm, Stockholm County, Sweden

[3] Department of Forestry, Food Technology, and Environmental Technology, South-Eastern Finland University of Applied Sciences, Kouvola, Kymenlaakso Region, Finland

[4] University of Eastern Finland School of Medicine, Institute of Biomedicine, Kuopio, North Savo, Finland

[5] Department of Obstetrics and Gynecology, C.S. Mott Center for Human Growth and Development, Wayne State University School of Medicine, Detroit, USA

[6] Center for Molecular Medicine and Genetics, Wayne State University School of Medicine, Detroit, USA

[7] Ovumia Kuopio, Kuopio, North Savo, Finland

[8] North Karelia Central Hospital, Joensuu, North Karelia, Finland

*Corresponding author: Jukka Kekäläinen, jukka.s.kekalainen@uef.fi

# Supplementary Results

### Identification of candidate FF factors associated with transcriptional activation

To understand which FF-derived factors may be selectively transferred to sperm alongside FF mRNA and potentially be involved in the transcriptional activation of above-mentioned CXCL genes in sperm, we performed gene ontology analysis for proteins previously reported to be present in FF [1]. In total 52 FF proteins were associated with Positive Regulation Of Gene Expression (GO:0010628, adj. P-value = 0.018). These proteins included, for example, PARK7, which is a positive regulator of androgen-receptor-dependent transcription [2] and has been predicted to play a role in fertilization [3]. Other identified proteins were *e.g.* GATA2 that is a well-known transcription factor with a crucial role already during early developmental stages [4] and FUBP3 that acts upon positive regulation of RNA polymerase II mediated transcription [5].

# Supplementary Methods

### Recording of sperm motility and statistical analysis of sperm performance

Sperm motility was recorded at 30 min and 180 min time points after the initiation of the sperm treatments using computer-aided sperm analysis (CASA; Integrated Semen Analysis System, ISAS, v. 1.2, Proiser, Valencia, Spain) with a negative phase contrast microscope (100 × magnification) and a capture rate of 100 frames $s^{-1}$. In the measurements, 1 $\mu$l aliquots from all sperm samples were added to Leja 4-chamber microscope slides (chamber height 20 $\mu$m, Leja, Nieuw-Vennep, the Netherlands). Then, we recorded two videos from two replicate aliquots per sample (n = 4 videos in total). The mean number of measured sperm cells per time point and per sample was 979 (± 42 SEM). After sperm motility measurements, cells were washed twice with 1ml of SW to wash out remnants of FF, pelleted, snap-frozen, and stored at -80°C until RNA extraction.

The effect of sperm treatments on sperm curvilinear swimming velocity (VCL) at both time points (30 min and 180 min) was tested in linear mixed models with the lmer function (lme4 R package, v. 1.1-28). In the models, sperm treatment (SW or FF) and replicate were used as fixed effects, and sperm donor was included as a random effect (1|sperm donor). Model assumptions were graphically verified using Q-Q plots and residual plots. The p-values for fixed effects were computed with the ANOVA function (lmerTest R package, v.3.1-3). All p-values presented are from two-tailed tests, with $\alpha = 0.05$.

### Sequencing library preparation and sequencing

Sequencing libraries were prepared separately for of each individually treated sperm subsamples within each three males (n = 9 male samples in total) and for the pooled FF sample using the SMARTer Stranded Total RNA-Seq Kit v3 – Pico Input Mammalian prep kit (Takara Bio, Mountain View, CA, USA) according to the manufacturer's instructions. The quality of purified libraries was assessed using the Qubit HS DNA assay kit (Invitrogen™, Thermo Fisher Scientific, Eugene, OR, USA) and the HS DNA kit (Agilent Technologies, Santa Clara, CA, USA). RNA-seq was performed on the Novaseq 6000 (2 × 150 bp reads, 50M reads per sample) sequencing platform in Novogene (Cambridge, UK).

### RNA-seq data preprocessing

FastQC (https://www.bioinformatics.babraham.ac.uk/projects/fastqc) was used to evaluate data quality. Read qualities and lengths were filtered using Trimmomatic using the following parameters: ILLUMINACLIP: 2:30:10, SLIDINGWINDOW: 4:10, and MINLEN: 35 [6]. Only reads containing a unique UMI sequence and having a unique alignment were kept using UMItools

(v. 1.1.2) and STAR aligner (v. 2.7.9a), respectively [7,8], aligning reads to the hg38 genome (Gencode v.38) with transcript annotation provided as a GTF file using a 2-pass mapping strategy. By this approach, it was possible to remove all the duplicates from the data. Read counts per gene generated by STAR were used in further analysis. For visualization, bam files were converted to TDF format in the Integrative Genomics Viewer.

**DE analysis workflow**

In this workflow, edgeR was used to import, organise, filter, and normalise the data. The count data and sample information were included into a DGE object for subsequent statistical analysis. The DGE object was created using DGEList function. Low expressed transcripts were filtered requiring CPM > 2.5 in three or more samples. Next, normalization factors were calculated using relative log expression (RLE) to ensure that the expression distributions of each sample are similar across the entire experiment. Then, limma with its voom method, linear modelling and empirical Bayes moderation was used to evaluate differential expression. Information of sperm donor, treatments, and library preparation batch was stored to a design matrix created using model.matrix function. Inclusion of the information of a sperm donor allowed considering individual-level variation. Then, a contrast matrix which defines the groups to be compared in the DE analysis, i.e., i) untreated (0h) versus SW-treated (3h), ii) untreated (0h) versus FF-treated (3h), and iii) SW-treated (3h) versus FF-treated (3h) was created using makeContrasts function. Next, heterogeneity of variation in the count data was removed using voom transformation. Finally, linear modelling (lmFit) and empirical Bayes moderation (eBayes) were carried out.

**Comparison on FF transcriptome and sperm DE genes**

FF gene names (raw count >20, table S1) were intersected with upregulated DE genes from each comparison (tables S2-4). The presence of a gene name in both lists was interpreted that the observed increase in the expression of that gene may be caused by internalization of mRNA from FF to sperm. Moreover, to systematically assess how FF transcript abundance (CPM) relates to expression changes (log2FC) of the same transcripts in FF-treated sperm, we calculated the Spearman correlation coefficients for untreated versus FF-treated sperm and SW-treated versus FF-treated sperm.

**Potential effect of other cell types on the sperm transcriptome**

We visualized the gene expression profiles of the upregulated sperm DEGs across multiple tissues provided by the GTEx project (figure S5). Some upregulated DEGs corresponding to fundamental regulators of cellular growth, such as *IGF2*, *JAG1*, and *NFKBIA* [9–11], are relatively ubiquitously expressed across tissues. In contrast, some DEGs are expressed only in a handful of tissues. These genes included e.g. *STAR* and *CYP11A1* that are present in adrenal gland, ovary, fallopian tube, and testis and thus could be observed in FF-treated sperm. In contrast, *CXCL2* is highly expressed in lung, liver and pancreas. The highest expression of both *CXCL3* and *CXCL8* was found in lung, minor salivary gland, and heart atrial appendage and *CXCL3* is additionally expressed in stomach. All of these tissues where the mentioned three CXCL genes are expressed are very unlikely to contaminate sperm samples. While *CXCL8* is also present in whole blood, no other blood markers were identified in sperm samples, supporting the view that blood cell contamination of our sperm samples is unlikely (figure S5B). Finally, the impact of potential immature sperm cell contamination was considered by visualizing the gene expression profiles of the upregulated sperm DE genes in cells representing pre-meiotic, meiotic, and post-meiotic stages of spermatogenesis (figure S6) [12]. This inspection revealed that the genes selected for nascent RNA capture validations (*CXCL3*, *CXCL8*, and *IGF2*) do not reflect RNA contamination from these spermatogenic cell types.

**ChIP-seq data preprocessing and visualization**

We retrieved a previously published human sperm ChIP-seq dataset including the following histone PTMs – H3K4me3, H3K4me1, and H3K27me3 – from the Sequence Read Archive or Gene Expression Omnibus (GSE15690 and GSE40195) [14,15]. Data quality was evaluated using the FastQC tool, and reads with low quality were removed using FastX-Toolkit (http://hannonlab.cshl.edu/fastx_toolkit/) with the following parameters: a minimum of 97% of all bases in one read was required to have a minimum Phred quality score of 10, and read had to be longer than 35bp and contain no adapter sequences. Reads were aligned to the hg38 genome (Gencode v.38) using the bowtie tool (v. 1.3.1) [16], allowing a maximum of two mismatches and two best category hits. Only one valid alignment per read was reported. Peak calling was performed using MACS2 (v. 2.1.1.20160309, with default parameters, including FDR cut-off (q-value of 0.01)). Broad or narrow peak parameter was selected according to Histone ChIP-seq Data Standards by the ENCODE project [17]. Peak coordinates were visualized using the Integrative Genomics Viewer.

# Supplementary Figures

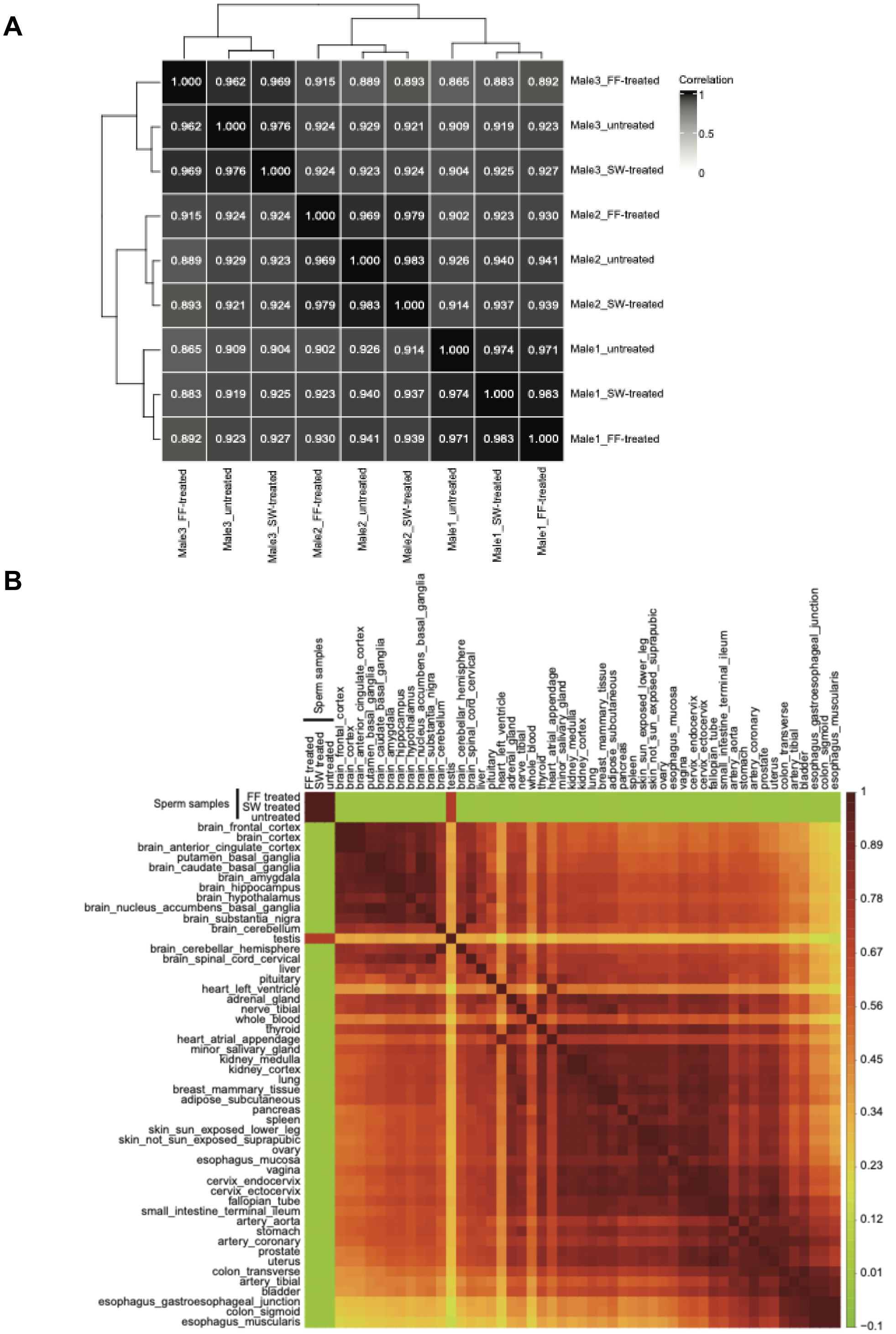


**Figure S1.** Quality assessments of sperm RNA-seq data. (A) Pairwise Spearman correlation heatmap of sperm RNA-seq samples calculated based on 11,777 genes that were inputted to differential gene expression analysis. (B) Pearson correlation coefficients of gene expression profiles between our sperm RNA samples and tissues provided by the GTEx project. Pearson correlation coefficients were calculated based on the top 1000 genes expressed in untreated mature sperm (RNA-seq data of the present study).

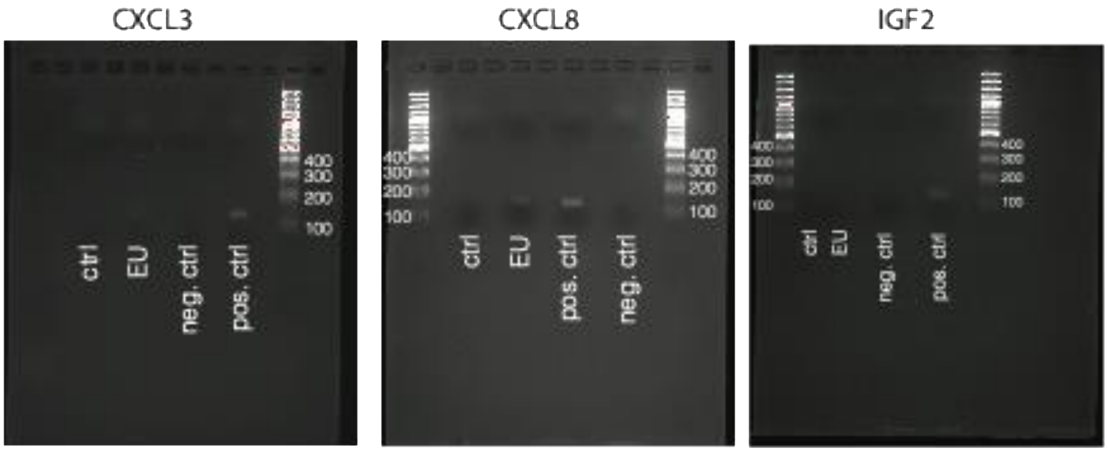


**Figure S2.** Second replicate of PCR analyses for *CXCL3*, *CXCL8*, and *IGF2* were performed using gene-specific exon spanning primers, and PCR products were separated using agarose gel electrophoresis. Ctrl = sperm samples treated with FF pool in the absence of the labelled nucleotide, 5-ethynyl uridine, to control any unspecific capture of RNAs, EU = sperm samples treated with FF pool in the presence of 5-ethynyl uridine to capture newly synthetized RNAs, pos. ctrl = cDNA synthetized from total RNA of an independent FF-treated sperm sample to show the expected size of PCR products and to account for potential non-specific PCR amplification that would show additional PCR products of unexpected size, and neg. ctrl = a water sample to exclude any non-specific amplification.

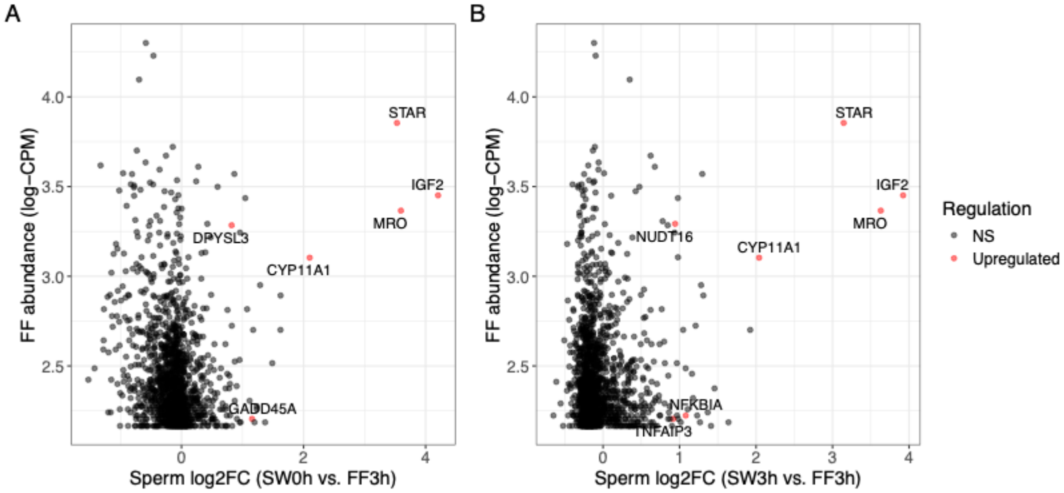


**Figure S3.** The association between follicular fluid transcript abundance (log-CPM) and their expression changes (log2FC) in follicular fluid -treated sperm in FF-treated vs. untreated (A) and in FF-treated vs. SW-treated sperm (B) comparisons. Transcripts that were identified as differentially expressed are highlighted in red. NS = not significant.

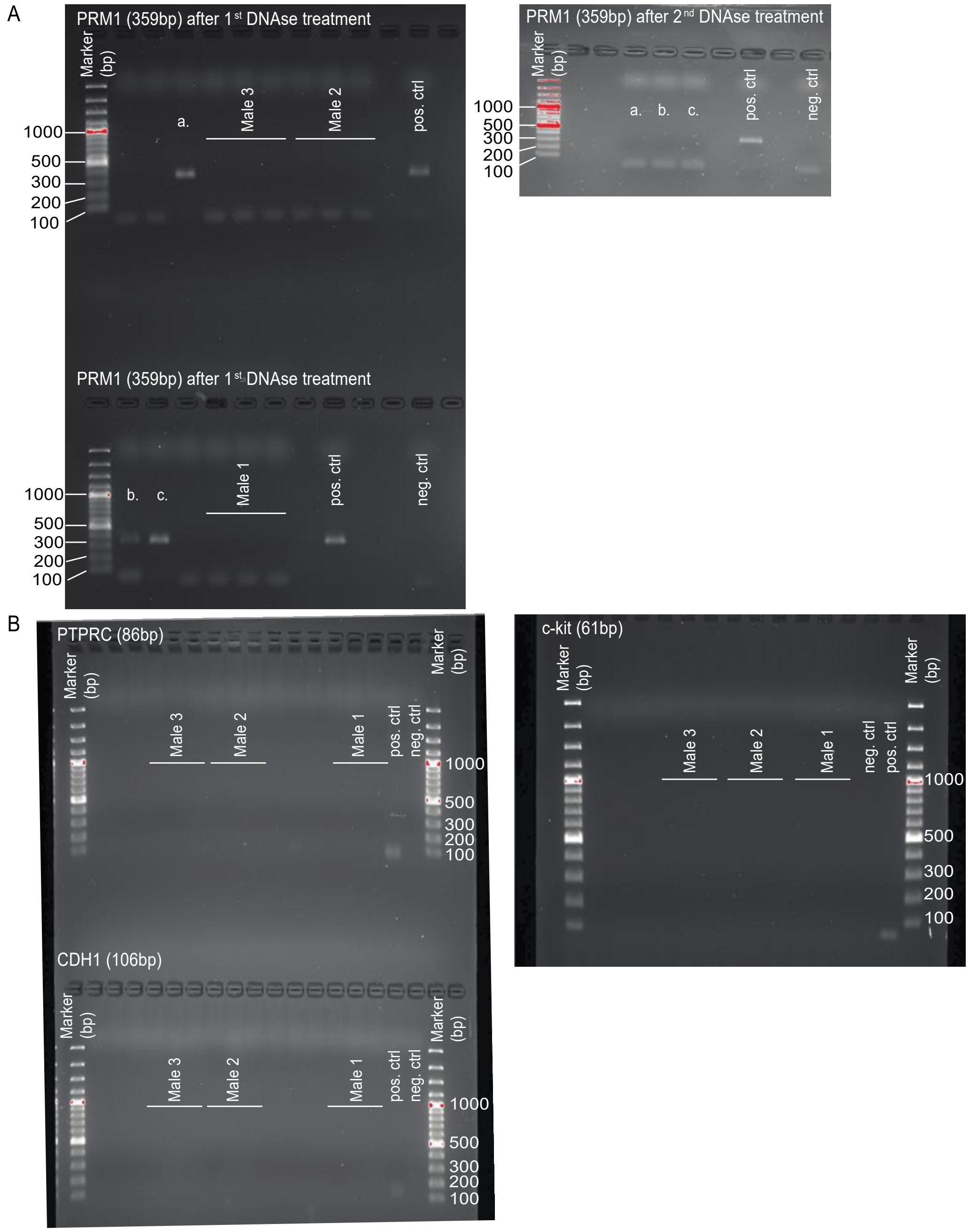


**Figure S4.** PCR analyses to confirm the absence of genomic DNA contamination and the absence of selected somatic and germ cell markers in sperm RNA samples as described in Goodrich et al, 2007 and Lambard et al, 2004, respectively. (A) PCR analysis of *PRM1* was performed using gene-specific exon spanning primers. PCR products were separated using agarose gel electrophoresis. Results after 1st DNAse treatment are shown on the left for the samples included in the final dataset (Male 1 – Male 3). Samples with DNA contamination are marked as a, b, and c. These samples were DNAse-treated 2nd time and successful removal of genomic DNA was confirmed (on the right). (B) PCR analyses of *PTPRC*, *CHD1*, and *c-kit* was performed using gene-specific exon spanning primers and PCR products were separated using agarose gel electrophoresis. Sperm RNA samples did not contain sequences of these genes.

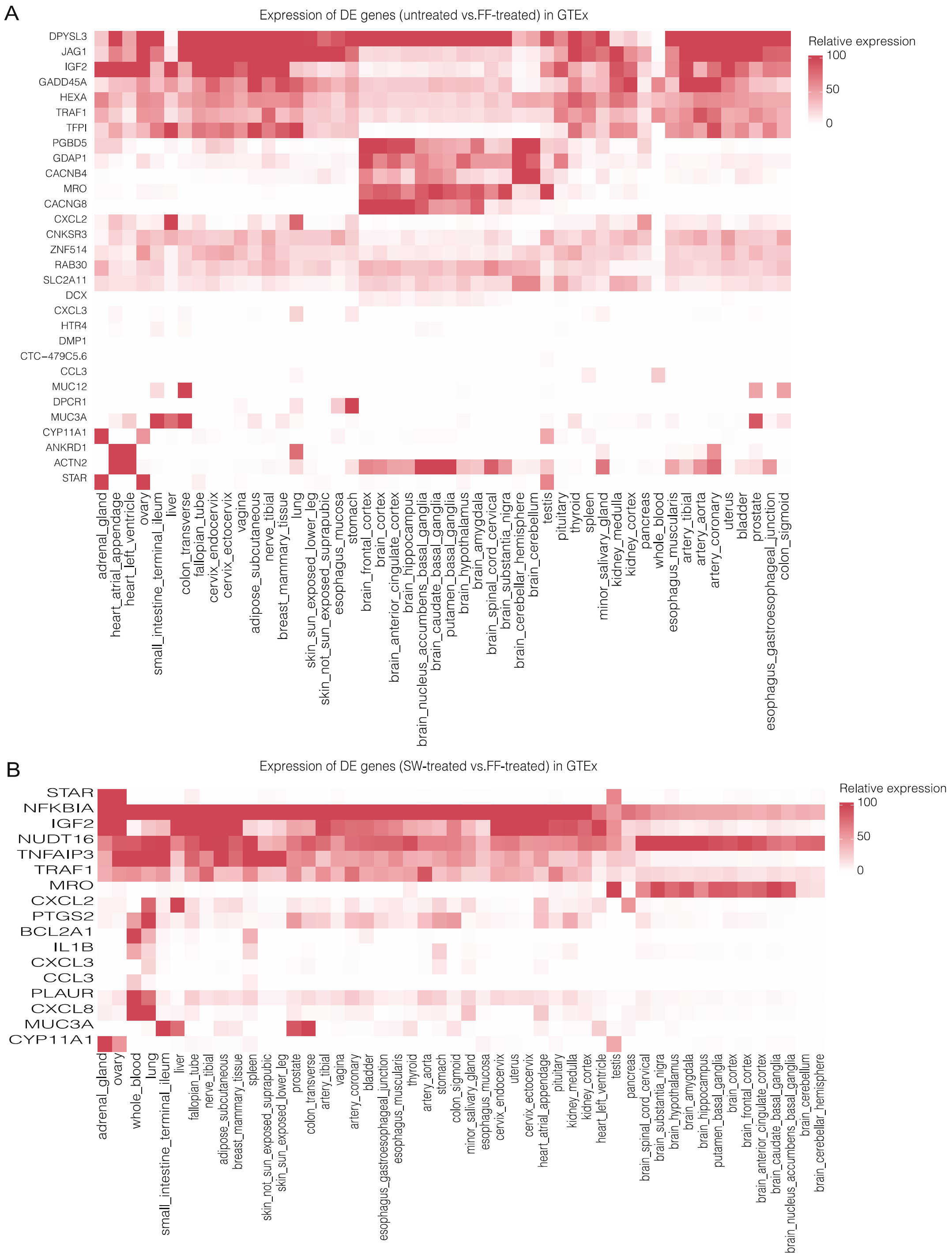


**Figure S5.** Expression of upregulated sperm DE genes in somatic cell data provided by GTEx. (A) DE genes in untreated vs. FF-treated sperm comparison. (B) DE genes in SW-treated vs. FF-treated sperm comparison.

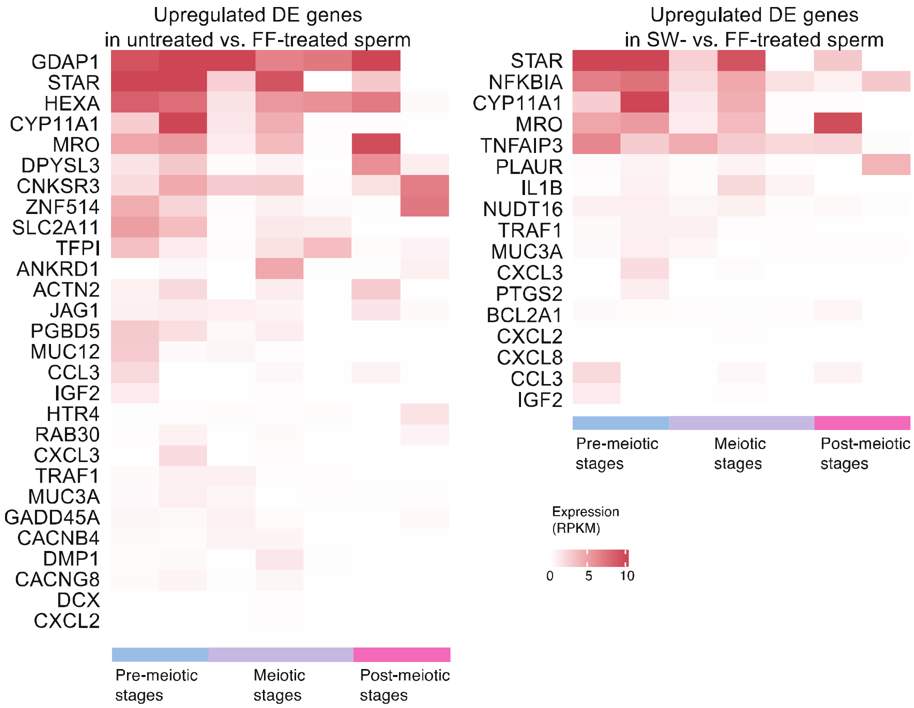


**Figure S6.** Expression of upregulated sperm DE genes in cells representing pre-meiotic, meiotic, and post-meiotic stages of spermatogenesis in the data published by Estill et al (2019). Upregulated DE genes in untreated vs. FF-treated sperm comparison are shown on the left and upregulated DE genes in SW-treated vs. FF-treated sperm comparison on the right. Two upregulated DE genes (*MUCL3* and *DPEP2NB*) were not captured in the published data; thus, they are absent in this plot.

## Supplementary References


1. Castillo J, Jodar M, Oliva R. 2018 The contribution of human sperm proteins to the development and epigenome of the preimplantation embryo. *Hum. Reprod. Update* **24**, 535–555. (doi:10.1093/humupd/dmy017)
2. Tillman JE, Yuan J, Gu G, Fazli L, Ghosh R, Flynt AS, Gleave M, Rennie PS, Kasper S. 2007 DJ-1 Binds Androgen Receptor Directly and Mediates Its Activity in Hormonally Treated Prostate Cancer Cells. *Cancer Res.* **67**, 4630–4637. (doi:10.1158/0008-5472.CAN-06-4556)
3. Cannarella R, Condorelli RA, Mongioì LM, La Vignera S, Calogero AE. 2020 Molecular Biology of Spermatogenesis: Novel Targets of Apparently Idiopathic Male Infertility. *Int. J. Mol. Sci.* **21**, 1728. (doi:10.3390/ijms21051728)
4. Castaño J *et al.* 2019 GATA2 Promotes Hematopoietic Development and Represses Cardiac Differentiation of Human Mesoderm. *Stem Cell Rep.* **13**, 515–529. (doi:10.1016/j.stemcr.2019.07.009)
5. Davis-Smyth T, Duncan RC, Zheng T, Michelotti G, Levens D. 1996 The Far Upstream Element-binding Proteins Comprise an Ancient Family of Single-strand DNA-binding Transactivators. *J. Biol. Chem.* **271**, 31679–31687. (doi:10.1074/jbc.271.49.31679)

6. Bolger AM, Lohse M, Usadel B. 2014 Trimmomatic: a flexible trimmer for Illumina sequence data. *Bioinformatics* **30**, 2114–2120. (doi:10.1093/bioinformatics/btu170)
7. Dobin A, Davis CA, Schlesinger F, Drenkow J, Zaleski C, Jha S, Batut P, Chaisson M, Gingeras TR. 2013 STAR: ultrafast universal RNA-seq aligner. *Bioinformatics* **29**, 15–21. (doi:10.1093/bioinformatics/bts635)
8. Smith T, Heger A, Sudbery I. 2017 UMI-tools: modeling sequencing errors in Unique Molecular Identifiers to improve quantification accuracy. *Genome Res.* **27**, 491–499. (doi:10.1101/gr.209601.116)
9. Paciolla M, Boni R, Fusco F, Pescatore A, Poeta L, Ursini MV, Lioi MB, Miano MG. 2011 Nuclear factor-kappa-B-inhibitor alpha (NFKBIA) is a developmental marker of NF-κB/p65 activation during in vitro oocyte maturation and early embryogenesis. *Hum. Reprod.* **26**, 1191–1201. (doi:10.1093/humrep/der040)
10. Grochowski CM, Loomes KM, Spinner NB. 2016 Jagged1 (JAG1): Structure, Expression, and Disease Associations. *Gene* **576**, 381–384. (doi:10.1016/j.gene.2015.10.065)
11. White V, Jawerbaum A, Mazzucco MB, Gauster M, Desoye G, Hiden U. 2018 IGF2 stimulates fetal growth in a sex- and organ-dependent manner. *Pediatr. Res.* **83**, 183–189. (doi:10.1038/pr.2017.221)
12. Estill MS, Hauser R, Krawetz SA. 2019 RNA element discovery from germ cell to blastocyst. *Nucleic Acids Res.* **47**, 2263–2275. (doi:10.1093/nar/gky1223)
13. Kuleshov MV *et al.* 2016 Enrichr: a comprehensive gene set enrichment analysis web server 2016 update. *Nucleic Acids Res.* **44**, W90–W97. (doi:10.1093/nar/gkw377)
14. Hammoud SS, Nix DA, Zhang H, Purwar J, Carrell DT, Cairns BR. 2009 Distinctive chromatin in human sperm packages genes for embryo development. *Nature* **460**, 473–478. (doi:10.1038/nature08162)
15. Hammoud SS, Low DHP, Yi C, Carrell DT, Guccione E, Cairns BR. 2014 Chromatin and transcription transitions of mammalian adult germline stem cells and spermatogenesis. *Cell Stem Cell* **15**, 239–253. (doi:10.1016/j.stem.2014.04.006)
16. Langmead B, Trapnell C, Pop M, Salzberg SL. 2009 Ultrafast and memory-efficient alignment of short DNA sequences to the human genome. *Genome Biol.* **10**, R25. (doi:10.1186/gb-2009-10-3-r25)
17. Luo Y *et al.* 2020 New developments on the encyclopedia of DNA elements (ENCODE) data portal. *Nucleic Acids Res.* **48**, D882–D889. (doi:10.1093/nar/gkz1062)
